# Biological Effects and Applications of Chitosan and Chito-Oligosaccharides

**DOI:** 10.3389/fphys.2019.00516

**Published:** 2019-05-07

**Authors:** Guiping Guan, Md. Abul Kalam Azad, Yuanshan Lin, Sung Woo Kim, Yun Tian, Gang Liu, Hongbing Wang

**Affiliations:** ^1^College of Bioscience and Biotechnology, Hunan Agricultural University, Changsha, China; ^2^Department of Animal Science, North Carolina State University, Raleigh, NC, United States; ^3^Hunan Province Key Laboratory of Animal Nutritional Physiology and Metabolic Process, Key Laboratory of Agro-ecological Processes in Subtropical Region, Institute of Subtropical Agriculture, Chinese Academy of Sciences, National Engineering Laboratory for Pollution Control and Waste Utilization in Livestock and Poultry Production, Changsha, China; ^4^University of Chinese Academy of Sciences, Beijing, China; ^5^Hunan Institute of Animal Husbandry and Veterinary Medicine, Changsha, China

**Keywords:** chitosan, chito-oligosaccharides, biological activity, application, microbiota

## Abstract

The numerous functional properties and biological effects of chitosan and chito-oligosaccharides (COS) have led to a significant level of interest, particularly with regard to their potential use in the agricultural, environmental, nutritional, and pharmaceutical fields. This review covers recent studies on the biological functions of COS and the impacts of dietary chitosan and COS on metabolism. The majority of results suggest that the use of chitosan as a feed additive has favorable biological effects, such as antimicrobial, anti-oxidative, cholesterol reducing, and immunomodulatory effects. The biological impacts reviewed herein may provide a new appreciation for the future use of COS.

## Introduction

The health and performance of modern intensively reared farm animals, such as poultry and swine, are promoted with the aid of various feed additives. The non-toxic linear polysaccharide chitosan made up of β-1-4 linked D-glucosamine and *N*-acetyl-D-glucosamine units, and its derivatives [chito-oligosaccharides (COS)] are comparatively novel and less frequently used as feed additives in animal nutrition. Chitosan, in varying levels of deacetylation, forms the basis of the common natural substance chitin, found in the exoskeletons of insects, crabs, and shrimps (Koide, 1998; Younes and Rinaudo, 2015). The biocompatibility and biodegradable properties of chitin have led to numerous reports of biomedical applications (Park and Kim, 2010). The major applications of chitin are in the production of monosaccharides, which constitute the primary dietary supplement in the United States, and in the relief of osteoarthritic pain (Aam et al., 2010). The chemical distinction between chitin and chitosan is based solely on the extent of the acetylation of the D-glucosamine units, with chitin being over 70% acetylated and chitosan under 30% acetylated. However, the application of chitin in living systems is comparatively limited by its insolubility in water, whereas chitosan is soluble in acidic solutions (Shahidi et al., 1999). Although chitosan is less commonly found in nature, its presence along with that of chitin in the cell walls and septa of filamentous fungi and yeast (Muzzarelli et al., 2012). The production of commercial chitosan via deacetylation of chitin involves the high-temperature treatment of chitin with a strong solution of sodium hydroxide (Singla and Chawla, 2001; Lemma et al., 2016). Approximately 150,000 tons of commercially applicable chitosan is produced annually by the conversion of chitin acquired as a by-product of seafood production (Fernandez and Ingber, 2014; Younes et al., 2014).

The de-polymerization of chitosan by acid hydrolysis, physical hydrolysis, and enzymatic degradation results in the production of COS (Lodhi et al., 2014). The weak glycosidic bonds in chitosan facilitate cleavage in the presence of hydrolyzing agents to generate chitosan oligomers incorporating various monomer units (Kim and Rajapakse, 2005). COS (also termed chitosan oligomers or chito-oligomers) are chitosan with an average molecular weight (MW) under 3.9 kDa and containing less than 20 monomer units per polymer chain (Lodhi et al., 2014). The low MW and the solubility of COS generates significantly more interest than the precursor species, hence with increasing commercial production.

The numerous biological properties of COS suggest a variety of possible uses in a wide range of areas, such as agriculture, cosmetics, food, and medicine (Xia et al., 2011). As no single type of chitosan or COS displays all of the observed biological activities, an increasing number of studies have aimed to examine the specific activities of this group of compounds. Furthermore, the various structures and physicochemical activities of chitosan derivatives and enzymatic products may furnish new biological activities or help generate a new understanding of previously known bioactive substances.

This review examines recent research focused on two important biological properties of chitosan and COS as applied to swine nutrition, thus providing a new understanding of these biological functions and pointing the way to the development of chitosan and oligosaccharides as swine-feed additives.

## Antimicrobial Properties and Regulation of Microbiota

The antimicrobial properties of COS and chitosan are well known. However, the exact mechanism of the antimicrobial activity of COS and chitosan is still unknown. Several studies have been suggested that the actual processes of antimicrobial activity could be occurred by changing the bacterial membrane permeability, cytoplasmic membrane barrier function or nutrient transport. In addition, the mechanism of antimicrobial activity mostly depends on MW, the degree of de-acetylation (DD), type of bacterium, pH, and the concentration of active compounds connected to chitosan and its derivatives (Jarmila and Eva, 2011; Guan et al., 2016).

The antimicrobial activity of chitosan or its derivatives usually depends on various factors including MW, DD, and other physicochemical characteristics along with microorganism type (Gram-negative or Gram-positive) (Liaqat and Eltem, 2018). COS has been broadly studied to improve the antimicrobial activities both *in vivo* and *in vitro*. The COS with positively charged can bind or absorb into the cell wall of microbes through negatively charged components of microorganisms present in the microbial cell. According to this concept, a study was conducted with 180 weaning pigs with average body weight were divided into five treatment groups and fed a control diet, treatment diet with COS (200, 400, or 600 mg/kg), and a diet with Colistin Sulfate (CSE) for 14 days. Results revealed that COS supplementation increases the population of *Bifidobacteria* and *Lactobacilli*, and decreases *S. aureus* in the cecum of the weaning pigs. The authors concluded that two possible mechanisms for the observed antimicrobial activity of COS. The first possible explanation was the positive charge on the NH_3_^+^ group of the COS glucosamine monomer interactions with a negatively charged microbial cell membrane. Secondly, COS may exert an indirect influence *via* increasing the populations of *Bifidobacteria* and *Lactobacilli* and the exclusion of *S. aureus* (Yang et al., 2012). Similarly, Kong et al. (2014) have found that dietary COS (0.5 g/Kg) supplementation in weaned Huanjiang mini-piglets increased the microbial population of *Bifidobacterium* spp., *Bifidobacterium breve*, *Faecalibacterium prausnitzii*, and *Lactobacillus* spp. in the ileum and colon. In addition, the number of *Fusobacterium prausnitzii*, *Methanobrevibacter smithii*, and *Roseburia* were increased in the colonic content of the dietary COS supplemented piglets. Furthermore, dietary COS supplementation decreased the microbial population of *Firmicutes* and *Streptococcus* in the ileum and colon, and *Bacteroides fragilis*, *Clostridium coccoides*, *C. leptum* subgroup., and *Eubacterium rectale* in the ileum, and *Escherichia coli* in the colonic content of the treated piglets. Of note, chemically modified COS has also been improved anti-microbial properties. For example, NO-releasing secondary amine-modified COS has been reported to readily penetrate the biofilm and associated with *Pseudomonas aeruginosa* and resulting in the effective killing of the *P. aeruginosa* biofilm through the effect of the released NO with the minimal inhibitory content (MIC) of 200 μg/mL (Lu et al., 2014).

In addition to antimicrobial activity, chitosan and COS have been shown to possess anti-fungal and anti-viral activities. However, with numerous studies attesting to the antifungal or anti-viral action of COS against a range of fungi and virus, the outcomes of studies on the anti-fungal or anti-viral properties of COS have been found somewhat inconsistent. These inconsistencies may be due to differences in the purity, quality, and properties of the COS used and/or the use of different microorganisms and methodologies. Although less potent than chitosan, COS has been shown to exhibit anti-fungal effects against several types of fungus including *Saccharomyces cerevisiae*, *Aspergillus niger*, *Trichophyton rubrum*, and *Candida* spp. with the MIC of 1.3 mg/mL (Seyfarth et al., 2008; Mei et al., 2015; Muanprasat and Chatsudthipong, 2017). Thus the potential clinical application of COS is desirable for its biocompatibility, biodegradability, and safety.

The gut microbiota is a very crucial factor that interacts with the host physiology and health (Niewold et al., 2010; Li et al., 2018); Wang et al., 2018). Alteration of gut microbiota plays an important role in host health, including vitamin synthesis, improve digestion, and promotion of angiogenesis and nerve function (Soler et al., 2014; Azad et al., 2018a; Wang et al., 2018). Chitosan and its derivatives have shown advantageous biological function in gut microbiota alteration. A study aimed to evaluate the effects of different levels of dietary COS (100, 200, and 400 mg/kg) supplementation during weaning period on growth performance, fecal shedding of *E. coli* and *Lactobacillus*, nutrient digestibility and small intestinal morphology. COS supplementation revealed an increase in the amount of fecal *Lactobacillus* along with a decrease in the amount of *E. coli* (Liu et al., 2008). Similarly, pigs were given 400 mg/kg supplementary COS in a study by Yang et al. (2012) also displayed enhanced populations of *Bifidobacteria* and *Lactobacilli* in the caecum on the 7th day after weaning compared to those weaned on the basal diet. On the 14th day after weaning, the same study also revealed a higher quantity of *Bifidobacteria* in the caeca of pigs given 600 mg/kg COS relative to those given the basal diet (Yang et al., 2012).

Chitosan or COS have been shown a potential activity on anti-obesity by altering the gut microbiota populations. In an obese animal model, (Egan et al., 2015) aimed to evaluate the effect of prawn shell derived chitosan (1000 ppm) in a pig model. The study was carried out with 125 days of age pigs (70 ± 0.09 kg body weight) were a fed basal diet or treatment diet (1000 ppm chitosan with basal diet) for 63 days. Results revealed that dietary chitosan supplementation reduced the populations of phylum Firmicutes in the colon and of *Lactobacillus* spp. in both the colon and the caecum, whereas the amounts of the *Bifidobacteria* genera in the caecum increased. Furthermore, sows fed with dietary chitosan exhibited lower feed intake and final body weight (Egan et al., 2015). Yan and Kim (2011) reported an enhanced blood lymphocyte count along with a decreased fecal population of *E. coli* in weaned pigs given 3 g/kg dietary COS (Yan and Kim, 2011), whereas Wan et al. (2017) reported that 100 mg/kg COS both enhanced the ileal *Bifidobacterium* population and decreased the *E. coli* and total bacteria populations of the colon and caecum (Wan et al., 2017). In earlier, corresponding results were obtained by Wang et al. (2009) who reported that dietary supplementation with COS (0.50%) decreased the populations of fecal *E. coli* in growing pigs, whereas the count of fecal *Lactobacillus* was unaffected. The glucosamine monomer unit of COS may interact with negative charges on the microbial cell membranes, resulting in the leakage of the cells’ internal constituents. In addition, COS has also been shown an indirect impact on the cell membrane by promoting *Bifidobacteria* and *Lactobacilli* populations which tend to exclude *S. aureus* (Yang et al., 2012).

A recent study examined the impact of dietary supplementation with COS of low MW (20,000 to 30,000 Da) on the gut microbiota of piglets and found significant differences in the composition of the gut microbiomes of the pigs, while pigs were given 50 mg/kg COS supplementation for 28 days of experimental periods along with the control group and in a group given antibiotics (Yu et al., 2017). Compared to the control group, the relative abundance of *Prevotella* increased, whereas the abundance of *Lactobacillus* decreased in both the COS supplemented group and antibiotic groups. In addition, the relative abundance of both *Succinivibrio* and *Anaerovibrio* were increased in the COS supplemented group and decreased in the antibiotics group (Yu et al., 2017). According to this study, microbial function prediction suggests that more pathways in cofactor and vitamin metabolism would be more enriched by the presence of COS compared than by the presence of antibiotics or the basal diet.

## Immunostimulatory, Immunoregulatory and Anti-Inflammatory Properties

The immune system is composed of innate immunity and adaptive immunity which plays an important role to prevent the foreign pathogenic substance from the body. In immune function enrichment, immunostimulating medicine, and nutraceuticals are of particular interest (Soler et al., 2014; Azad et al., 2018b; Liaqat and Eltem, 2018). Dietary COS has been demonstrated effective and promising immunostimulator activities in both *in vivo* and *in vitro* models. According to Zhang et al. (2014), the immunostimulatory properties of COS may occur via interaction with membrane receptors on the macrophage surface and depend on toll-like receptor 4 (TLR4). A significant dose- and MW dependent immunoregulatory responses have been observed in the presence of COS with MWs of 3 and 50 kDa. The presence of COS can boost the expression of the gene molecules essential to the NF-κB and AP-1 pathways and trigger protein phosphorylation in the RAW264.7 macrophage. In this study, COS with a MW of 3 kDa demonstrated more promise as a new treatment for immune suppressive conditions with potential application in vaccines (Zhang et al., 2014). This also suggests the potential use of COS as a component of functional food designed to combat diet-related and age-related conditions. The clinical testing of immunostimulation by orally administered COS has already conducted.

Immunomodulatory feed additives such as chitosan or its derivatives may act as alternatives to antimicrobial growth promoters in pig production. Therefore, (Yin et al., 2008) designed an experiment of the pig model to examine the immunoregulatory function of early weaned piglets. They fed 0.025% of dietary COS along with 0.2% of galacto-mannan-oligosaccharides (GMOs) or 0.11% of lincomycin. After the end of 2 weeks experimental period, the results revealed that the weaning challenge led to reduced levels of serum antibodies and cytokines, the administration of 250 mg/kg COS resulted in higher expression of the IL-1β gene in the lymph nodes and jejunal mucosa and higher concentrations of interleukins IL-2, IL-6, and IL-1β and immunoglobulins IgA, IgG, and IgM in the serum. Hence, the authors concluded that dietary COS promotes the cell-mediated immune reaction in early weaned piglets by regulating the generation of antibodies and cytokines (Yin et al., 2008). Similarly, Wan et al. (2017) indicated that the administration of 100 mg/kg dietary COS enhanced superoxide dismutase (SOD) and catalase (CAT) activities, total antioxidant capacity and the serum levels of IL-6, IgG, and TNF-α. Additionally, a 26.59% decrease in the concentration of serum malondialdehyde (MDA) was noted for the pigs given dietary COS. A recent study by Li et al. (2017) reported that the dose-dependent dietary chitosan (100, 500, 1000, and 2000 mg/kg feed) enriched the linear or quadratic levels of prostaglandin E2, leukotriene B4 and arachidonic acid in piglets given dietary chitosan. Linear or quadratic enhancements in the activity of serum cytosolic-phospholipase A2 were also observed, as well as a quadratic enhancement in the activity of COX-2 and a linear enhancement in the activity of 5-lipoxygenase. These observations suggest that arachidonic acid metabolism is modulated by chitosan in a dose-dependent manner, which may partly explain why chitosan influences the immune function of weaned piglets through the AA pathway (Li et al., 2017).

Sun et al. (2009) evaluated the effects of chitosan (250 mg/kg, MW = 10^3^ to 10^4^), GMOs (2000 mg/kg) on the growth performance, serum immune parameters of 28-day weaned piglets challenged with pathogenic *E. coli*. Feed gain ratio and IgA, IgG, and IgM levels were increased in an *E. coli* challenged model by dietary COS supplementation. Similarly, Xiao et al. (2013, 2014) investigated the effects of dietary COS on growth performance, jejunal morphology, jejunal mucosal secretory IgA, occludin, claudin-1, and TLR4 expression in weaned piglets challenged by enterotoxic *E. coli*. A total of thirty piglets were fed a corn-soybean diet as a control diet, 50 mg/kg chlortetracycline, or 300 mg/kg COS. After 21 days experimental period, the findings showed that dietary COS and chlortetracycline reduced FCR, villus height, crypt depth, and the TLR4mRNA expression but increased the villus length, villus length/crypt depth, and goblet cells. In addition, the secretory IgA was observed higher in the dietary COS group compared with the other groups. Therefore, the authors concluded that chitosan showed similar effects with antibiotics in promoting the growth and reducing the intestinal inflammation in weaning piglets. Later, the same authors used a similar model to evaluate the effects of dietary COS on intestinal inflammation. The finding supports the previous work, and additionally, dietary supplementation with 300 mg/kg COS improved the mRNA expression of IL-1β and IL-6 in the jejunal mucosa (Xiao et al., 2013, 2014). Thus it proves that as a feed additive, dietary chitosan may influence different mechanism to alleviate inflammation in weaning piglets.

Several studies have examined the impacts of dietary chitosan supplements on antioxidative enzymes and stress hormones, and on humoral and cellular immune function in weaned piglets. Li et al. (2013) found a dose-dependent quadratic enhancement in the levels of serum IgG, and a dose-dependent linear or quadratic enhancement in the levels of serum specific ovalbumin IgG (Fan et al., 2013; Li et al., 2013). However, the levels of serum IgA and IgM were unaffected (Fan et al., 2013). The same authors reported a linear dose-dependent reduction in the levels of serum adrenocorticotropic hormone along with a dose-dependent linear or quadratic reduction in the levels of serum cortisol. Enhancements in the levels of CAT, SOD, and serum glutathione peroxidase with increasing chitosan were also noted, demonstrating that dietary chitosan enhances the activity of antioxidative enzymes and reduces weaning stress in piglets (Li et al., 2013). According to Huang et al. (2016) the impacts of dietary COS (300 μg/kg) on intestinal inflammation and the NF-κB signaling pathways in an LPS-challenged piglet model demonstrated the significant easing of LPS-induced intestinal injury. Furthermore, dietary COS reduced serum concentrations of IL-6, IL-8, and TNF-α, decreased intestinal levels of pro-inflammatory cytokine mRNA and increased levels of anti-inflammatory cytokine mRNA relative to the control group. The protein expression of IKKα/β, IκB, and phospho-NF-κB p65 also reported for the LPS-challenged piglets in the COS diet group (Huang et al., 2016). Therefore, dietary chitosan or its derivatives may play a crucial role in oxidative stress, intestinal inflammatory response, as well as by the inhibition of NF-κB signaling pathways under an inflammatory stimulus.

The anti-inflammatory properties of COS have been widely reported in the view of the potentially damaging effects of a disproportionate and protracted inflammatory response in a range of illnesses (Ngo et al., 2011). Efforts to explain the anti-inflammatory properties of chitosan and COS have focused on numerous potential mechanisms, for example, the acid hydrolysis of chitosan to glucosamine hydrochloride, sulfate, phosphate or other salts by salt conversion. Alternatively, the suppression of LPS-induced inflammatory gene expression by COS has been linked to the decreased nucleus translocation of the nuclear factor kappa-light-chain-enhancer of activated B cells (NF-κB) (Li et al., 2011). Hence, COS effectively reduces both inflammations due to infection by enterotoxigenic *E. coli* and LPS-induced vascular endothelial inflammation (Liu et al., 2016; Xiao et al., 2016). Moreover, COS significantly reduces the LPS-induced phosphorylation of p38 mitogen-activated protein kinase (MAPK) and extracellular signal-related protein kinase 1/2 and may hinder the activation of NF-κB and activator protein-1 (AP-1). The results of a study by Xiao et al. (2013) in weaned piglets challenged with enterotoxigenic *E. coli* revealed that the dietary COS (0.03%) and antibiotic (chlortetracycline) had similar beneficial effects in reducing intestinal inflammation and promoting growth. Similar to an antibiotic, dietary COS supplementation increased, the concentration of intra-epithelial lymphocytes, goblet cells, villus length, villus length to crypt depth ratio, occluding protein and secretory IgA protein expression, decreased TLR4 mRNA expression. Finally, the authors concluded that COS has the potential against inflammation Xiao et al. (2013). Furthermore, dietary supplementation of COS can activate the expression of inducible nitric oxide synthase and cyclooxygenase-2 (COX-2) induced by TNF-α in synoviocytes was inhibited via COS-reduced AMPK activation, resulting in the attenuation of synovial inflammation (Kunanusornchai et al., 2016).

Chronic inflammation of the gut is involved in various forms of inflammatory bowel disease (IBD), such as Crohn’s disease and ulcerative colitis (Herfarth, 2013; Hill, 2014; Guan and Lan, 2018; Hong and Piao, 2018; Zhang Y. et al., 2018). The occurrence of IBD has steadily increased in certain parts of the world in the last 40 years, perhaps as a result of changing dietary practices, including the preference for low-fiber diets (Rose et al., 2007; Umakanthan et al., 2016; Weichselbaum and Klein, 2018; Zhang X. et al., 2018). The known anti-inflammatory properties of COS have therefore prompted scientists to examine its potential as an adjuvant treatment for inflammatory illnesses. For example, tissue damage and reduction in colon length have been ameliorated and the inflammation of the colonic mucosa has been prevented in mice given COS orally, suggesting the potential application of COS as a functional food for individuals with IBD (Azuma et al., 2015). A recent study was carried out to investigate the effect of dietary COS supplementation on pig growth. The results revealed that the pigs consumed COS for 21 days increased average daily body weight gain compared to those in the control group. Besides the improvement of the activities of superoxide dismutase (SOD), catalase (CAT), and total antioxidant activity dietary COS also increased the IL-6, TNF-α, and IgG concentrations in the serum. In addition, dietary COS were found to increase the total bacterial populations of *Bifidobacterium* in the ileum and colon. Finally, the outcomes suggested that the growth of pigs during weaning can be accelerated by dietary COS supplementation because dietary COS can enhance the antioxidant and immune properties, as well as intestinal development (Wan et al., 2017). Therefore, the potential application of dietary COS should be further investigated as an anti-inflammatory compound in animal diets, food, and pharmaceutical industries.

## Effects on Performance, Digestion and Intestinal Structure in Swine Nutrition

The effectiveness and nutritional significance of COS as an animal-feed additive are summarized in Table 1. Importantly, a number of beneficial impacts have been noted during the weaning stage—a vital time for growing pigs, during which they are subject to environmental, immunological and nutritional pressures that frequently exert detrimental effects on a range of metabolic functions, resulting in digestive illnesses, diarrhea, limited growth and increased mortality (Swiatkiewicz et al., 2015; Oliveira et al., 2017; Zhao et al., 2017). For instance, (Liu et al., 2008) performed a study aimed to evaluate the effect of dietary COS supplementation (100, 200, and 400 mg/kg) on growth performance, fecal shedding of *E. coli* and *Lactobacillus*, nutrient digestibility, and small intestinal morphology in weaned pigs. Results revealed that dietary COS (100 and 200 mg/kg) supplementation improved average body weight gain (BWG), beneficial effects on feed intake (FI), and feed conversion ratio (FCR) compared to the control pigs. Additionally, dietary COS supplementation decreased diarrhea scores and increased *Lactobacillus* counts than those from control pigs (Liu et al., 2008). Yang et al. (2012) found the pigs were given dietary COS from the first to seventh day after weaning to have increased ADG and ADFI values compared to the control group. Although dietary supplementation with COS (400 mg/kg or 600 mg/kg) from the first to the 14th day after weaning resulted in enhanced ADG and gain/feed ratio, no effect on the crypt depth and villous height of the ileum, jejunum or duodenum was observed (Yang et al., 2012). The aim of an experiment by Zhou et al. (2012) was to assess the growth performance, nutrient digestibility, and incidence of diarrhea in weaned pigs. According to their purposes of the study, a total of 120 weaned pigs (21 ± 1 days of age) with average body weight (7.10 ± 0.48 kg) were divided into four dietary treatment groups; (a) CON, basal diet, (b) ANT: basal diet with antibiotic treatment, (c) COS1, basal diet with 1 g/kg COS, and (d) COS2, basal diet with 2 g/kg COS. At the end of study results showed that the higher addition of COS (2 g/kg) enhanced the total tract apparent digestibility of dry matter and nitrogen and growth performance and reduced the incidence of diarrhea. However, digestibility and growth performance were both reduced for the pigs’ given dietary additions of antibiotics (Zhou et al., 2012).

**TABLE 1:** Effects of dietary chito-oligosaccharides (COS) supplementation on the performance of pigs.

**Concentration of COS in the diet**	**Animals**	**Results**	**References**
1000 ppm	Females pigs, 70 kg ± 0.90	Reduced feed intake and final body weight (*P* < 0.001), lowered ileal digestibility of dry matter (DM), gross energy (GE; *P* < 0.05), and reduced coefficient of apparent total tract digestibility (CATTD) of gross energy and nitrogen (*P* < 0.05)	Egan et al.,2015
300 μg/kg	Similar body weight, 28 days	Lowered serum concentrations of tumor necrosis factor alpha (TNF-α), interleukin (IL) 6, and IL-8 (*P* < 0.05)	Huang et al.,2016
100, 500, 1000, or 2000 mg/kg	Average body weight of 7.6 kg, 28 days	Improved serum NO concentrations and iNOS activity (*P* < 0.05). Enhanced the expressions of iNOS mRNA in the jejunum and ileum	Li et al.,2017
100, 500, 1000, and 2000 mg /kg	Average body weight of 7.6 kg, 28 days	Improved the serum immunoglobulin G (IgG) and secretory immunoglobulin A (sIgA; *P* < 0.05). Decreased serum concentrations of soluble CD4 and soluble CD8 (*P* < 0.05)	Li et al.,2013
100, 500, 1000, and 2000 mg /kg	Average body weight of 7.6 kg, 28 days	Increased serum AA, prostaglandin E2, and leukotriene B4 contents, improved the activities of serum cytosolic-phospholipase A2, 5-lipoxygenase activity and cyclooxygenase-2 activity	Li et al.,2017
100, 200, and 400 mg/kg	4.72 ± 0.23 kg; 16 days	Improved ADG, ADFI and G: F, increased (*P* < 0.05) the digestibility of DM, Ca, and P, incidence of diarrhea and decreased diarrhea scores	Liu et al.,2008
40%	4.9 ± 0.3 kg, 17 ± 3 day	Reduced the incidence of diarrhea, the growth performance of *Escherichia coli*-challenged pigs than that of unsupplemented pigs challenged with *E. coli*	Liu et al.,2010
100 mg/kg	5.33 ± 0.369 kg, 17 days	Positive effect on the feed conversion ratio (FCR) and incidence of diarrhea	Oliveira et al.,2017
20 g/kg	60.3 ± 2.1 kg, 14 days	No influence (*P* > 0.05) on nutrient digestibility or N utilization	O’Shea et al.,2011
250 mg/kg	8.09 ± 1.87 kg, 28 days	Increased serum concentrations of IgG, IgA, IgM, IL - 6, I L -2, and IL – l β (*P* < 0.05), no difference in average daily gain or feed intake	Sun et al.,2009
75, 150, or 225 mg/kg	5.68 ± 0.07 kg, 18 days	Increased the digestibility of crude protein, crude fat, ash, calcium, and phosphorus, and also villus height and villus height/crypt depth ratio (*P* < 0.05)	Suthongsa et al.,2017
100 mg/kg	7.78 ± 0.09 kg, 28 days	Increased average daily body weight gain and digestibility of crude protein, ash, fat, dry matter, and gross energy (*P* < 0.05)	Wan et al.,2017
100 mg/kg	Multiparous sows	Enhanced the fetal survival rate and size (crown-to-rump length) of multiparous sows, increased the survival rate of piglets per fetus and the average weight of piglets	Wan et al.,2016
300 mg/kg	5.65 ± 0.27, 21 days	Elevated *(P* < 0.05) the mRNA expression of IL-1b, and IL-6 in the jejunal mucosa, decreased the calprotectin levels, and TLR4 protein expression	Xiao et al.,2014
300 mg/kg	21 days	Reduced feed conversion ratio, villus width, and crypt depth (*P* < 0.05), increased villus length, villus length/crypt depth, and goblet cells (*P* < 0.05)	Xiao et al.,2013
30 mg /kg	Pregnant sows with the same parturition history	Increased villus length and ratio of villus length to crypt depth in the ileum and jejunum (*P* < 0.01), stimulate plasma glutathione peroxidase activity (*P* < 0.01)	Xie et al.,2016c
30 mg/kg	Pregnant sows with same parturition history	Enhanced daily gain and weaning weight (*P* < 0.05), and the concentration of amino acids in sow milk (*P* < 0.05). Increased the expression of PEPCK-C, PEPCK-M, and G6Pase in the liver of piglets	Xie et al.,2015
30 mg /kg	Pregnant sows with a similar parturition number	Promoted the plasma and hepatic cholesterol accumulation and up-regulated the mRNA level of negative-regulated element period 1, reduced the circadian locomotor output cycles kaput, and brain muscle Arnt-like 1 of the suckling piglets	Xie et al.,2016b
30 mg /kg	Pregnant sows with the same parturition history	Increased plasma total SOD and the expression of some antioxidant genes in the placenta, reduced plasma MDA (0.05 < *P* < 0.10) and pro-inflammatory cytokines (*P* < 0.05)	Xie et al.,2016c
30 mg/kg	7.86 ± 0.22 kg; 21 days	No influence (*P* > 0.05) on growth performance, reduced villus height (*P* < 0.05) and villus height: crypt depth (*P* < 0.05) in the ileum	Xiong et al.,2015
100, 500, 1000, and 2000 mg/kg	11.56 ± 1.61 kg; 35 days	Improved average daily gain (ADG), apparent digestibility of crude protein (CP) and digestibility of dry matter (DM; *P* < 0.05)	Xu et al.,2014
**Concentration of COS in the diet**	**Animals**	**Results**	**References**
100, 500, 1,000, and 2,000 mg/kg	11.56 ± 1.61 kg; 35 days	Enhanced average body weight gain (BWG), increased the concentration of serum GH, the villus height of jejunum and ileum, and the ratio of villus height to crypt depth in duodenum, jejunum, and ileum	Xu et al.,2013
3 mg/kg	5.97 ± 0.46 kg; 21 days	Decreased the *E. coli* numbers, increased the lymphocyte count in blood. No influenced on nutrient digestibility, and blood characteristics	Yan and Kim,2011
200,400, or 600 mg/kg	5.98 ± 0.04 kg; 21 ± 3 days	Increased ADG, ADFI and G:F (*P* < 0.05), reduced diamine oxidase (DAO) in the serum. Enriched the concentration of *Bifidobacteria* and *Lactobacilli* (*P* < 0.05), lowered the population of cecal *Staphylococcus aureus* (*P* < 0.05)	Yang et al.,2012
30 mg/kg	7.82 ± 0.21 kg; 25 days	Increased serum IgG, urea nitrogen contents and serum calcium (*P* < 0.05). Improved the concentration of short chain fatty acid (SCFA) in caecal and colonic digesta	Yang et al.,2016
100 mg/kg	Male pigs; 15 ± 1 kg	Reduced oxygen consumption and the net absorption of dietary AA into the portal vein	Yin et al.,2010
250 mg/kg	5.6 ± 0.51 kg; 15 day	Increased (*p* < 0.05) IL-1β gene expression in jejunal mucosa and lymph nodes, and serum levels of IL-1β, IL-2, IL-6, IgA, IgG, and IgM	Yin et al.,2008
50 mg/kg	Average weight of 6 kg; 21 day	Increased the relative abundance of *Succinivibrio* and *Anaerovibrio*, decreased the ratio of *Lactobacillus*	Yu et al.,2017
160 mg/kg	8.24 ± 0.88 kg; 28 days	Improved (*P* < 0.01) average daily gain and feed conversion ratio, increased peripheral blood lymphocyte proliferation and serum-specific ovalbumin antibody level (*P* < 0.01)	Zhao et al.,2017
1, 2 g/kg	7.10 ± 0.48 kg; 42 days	Improved total tract apparent digestibility (TTAD) of dry matter (DM) and nitrogen (N; *p* < 0.05)	Zhou et al.,2012

Chitosan have the ability to enhance their bioavailability in the extraintestinal tissues by reducing oxygen consumption, as well as the dietary amino acid (AA) absorption into the portal vein in young pigs (Yin et al., 2010). Research has consistently demonstrated the enhanced digestibility of the ileal contents, enhanced adsorption capacity, and increased cell division, thus clearly indicating the potential applicability of COS as a dietary additive in raising the efficiency of the digestive process and stimulating nutrient adsorption (Suthongsa et al., 2017). A study conducted by Xu et al. (2013) examined the growth performance, small intestinal structure and serum growth hormone (GH) concentration of weaned pigs, the dietary administration of COS (100, 500, 1,000 and 2,000 mg/kg) enhanced BWG quadratically. Moreover, the dietary administration of COS led to quadratic increases in the serum GH concentration, the ileum and jejunum villus heights, and the villus height to crypt depth ratios of the ileum, jejunum, and duodenum (Xu et al., 2013). This study concluded that the enhanced growth performance of the following dietary administration of COS could be the direct result of enhanced serum GH levels and the improved morphology of the small intestine (Xu et al., 2013). These conclusions were substantiated by the same authors in another investigation in which they examined the positive impacts on the growth of weaned pigs given 1 g/kg or 2 g/kg dietary COS. The study suggested that the enhanced growth of the weaned pigs given dietary COS could also link to the enhanced digestibility of calcium, phosphorus, crude protein, and dry matter and enhanced levels of amylase in the jejuna (Xu et al., 2014). Nevertheless, the above outcomes have been disputed by other studies. For example, (O’Shea et al., 2011) observed no effect on nutrient digestibility or nitrogen utilization following chitosan consumption. Similarly, the administration of 0.30% dietary COS in a study by Yan and Kim (2011) had no impact on nutrient digestibility or growth performance.

Research evidence has found that dietary low-dosage of COS with high purity not only experienced on growth-enhancing effects but also displayed a tendency toward decreased villus height in the jejunum or duodenum (Xiong et al., 2015; Yang et al., 2016). A recent study was aimed to evaluate the effects of low-dosage COS (30 mg/kg, MW = 800–2000 Da, water solubility = 99%) on intestinal mucosal AA profiles and alkaline phosphatase (ALP) activities, and serum biochemical variables in weaned piglets. For these purposes of the experiment, a total of 24 piglets (25 days of age) assigned into two groups (control group and treatment group) for 14 days. The results demonstrated that the dietary COS supplementation increased serum IgG, calcium, and serum urea nitrogen contents. Moreover, dietary COS increased the contents of some AA in the mucosa of jejunum and ileum, ileal mucosal ALP activity, and luminal short-chain fatty acids (SCFA) in the cecum and of the weaned piglets (Yang et al., 2016). Earlier, the same authors used similar dietary COS to evaluate the intestinal morphology, immune response, antioxidant capacity, and intestinal barrier function of weaned piglets. Results showed that dietary COS increased stomach pH, IL-6 (duodenum, jejunum, and ileum), and secretory IgA (duodenum and ileum), and reduced villus height and villus height to crypt depth ratio in the ileum. Thus the outcomes suggest that supplemental COS at low dosage may lead to immunological and oxidative stress in the small intestine and damage the integrity of the intestinal barrier in weaned piglets (Xiong et al., 2015; Guan et al., 2016).

The quantity of studies assessing the effects of dietary COS on weaned piglets far outnumbers those dealing with pigs or sow’s coming to the end of their growth phase. In a study in which dietary chitosan was administered to sows with approximate body masses of 70 kg, (Egan et al., 2015) found reductions in FI, final body weight, the ileal digestibility of dry matter, gross energy, and the coefficient of apparent total tract digestibility of the gross energy of nitrogen relative to the control group (Egan et al., 2015). Furthermore, (Xie et al., 2015, 2016b, 2016c) investigated the effects on plasma glucose levels in suckling piglets following the dietary administration of COS (30 mg/kg) to maternal sows during gestation and lactation. In one of their studies, the daily gain and weaning weight of the piglets were enhanced, and AA concentration was increased in sow milk (Xie et al., 2015). Higher plasma glucose levels and lower hepatic glycogen levels also noted in the piglets of the COS-fed sows relative to those of the control group. In another study, the piglets of sows given dietary COS displayed increased villus length, an increased villus length to crypt depth ratio in the jejunum, and ileum and increased activity of plasma glutathione peroxidase (Xie et al., 2016a). The mRNA levels of the transcription-translation negative feedback element period 1 were enhanced and the mRNA levels of the positive feedback elements, the gene encoding the basic helix-loop-helix-PAS transcription factor (CLOCK) and brain and muscle Arnt-like protein-1 were reduced (Xie et al., 2016c).

It is worth noting that the mRNA expression of genes for certain antioxidants was enhanced in the placenta following the administration of dietary COS, whereas the levels of pro-inflammatory cytokines decreased. Further investigation indicated that the administration of dietary COS triggered the mTOR signaling pathway and enhanced the expression of AA transporters in the placenta (Xie et al., 2016c). These results were backed up by Wan et al. (2016) in their examination of the reproductive performance and gene expression of specific biochemical markers in the fetuses and placentas of sows following dietary COS (100 mg/kg) administration after 35 days of gestation. In addition, a 100-mg/kg dose of dietary COS supplementation after 35 days of gestation considerably increased the fetal survival rate and size. Furthermore, the number of viable piglets born per litter and the average weights of the live piglets at birth also increased considerably following dietary COS administration during gestation (Wan et al., 2016). Therefore, dietary COS is effective in increasing the growth performance, improving intestinal structures, and utilization of dietary protein by pigs.

## Conclusion

Chitosan and COS display a significantly broad range of biological properties that confer a possible potential for a variety of commercial uses. This review demonstrates that the use of chitosan and its derivatives as a pig-feed additive provides positive antimicrobial, anti-oxidative, immunoregulatory, and blood cholesterol limiting effects. Nevertheless, it is important to realize that various structures of chitosan and COS displayed different biological properties, with no single type of chitosan displaying the full range of properties. The majority of the studies have demonstrated the beneficial effects of chitosan, such as enhanced nutrient digestibility and enhanced the growth performance (in terms of FCR and/or BWG) in weaned piglets. Nevertheless, the molecular mechanisms of these bioactivities and the precise influences of the physicochemical properties of these substances on their various bioactivities remain to be understood. In the majority of published studies, the available experimental data suggest that the growth-enhancing effects of chitosan are comparable to those of dietary antibiotics. Hence, chitosan is a promising and effective alternative to antibiotics.

## Author Contributions

GG and GL initiated the idea and the outline of this review manuscript. GG and MA wrote the manuscript. MA, SK, YL, YT, and HW provided intellectual oversight, suggestions, and editing. MA revised the manuscript critically for intellectual content. All authors read and approved the final manuscript.

## Conflict of Interest Statement

The authors declare that the research was conducted in the absence of any commercial or financial relationships that could be construed as a potential conflict of interest.
